# Association between symptoms of COVID-19 infection and adverse maternal-perinatal outcomes in pregnant women at a referral hospital

**DOI:** 10.17843/rpmesp.2023.401.11205

**Published:** 2023-03-28

**Authors:** Kevyn Angulo-Fernandez, Adrian Olivera-Rojas, Benoît Mougenot, Percy Herrera-Añazco

**Affiliations:** 1 Universidad Peruana de Ciencias Aplicadas, Lima, Peru. Universidad Peruana de Ciencias Aplicadas Universidad Peruana de Ciencias Aplicadas Lima Peru; 2 Center of Excellence in Economic and Social Research in Health, Faculty of Business Sciences, Universidad San Ignacio de Loyola, Lima, Peru. Universidad San Ignacio de Loyola Center of Excellence in Economic and Social Research in Health Faculty of Business Sciences Universidad San Ignacio de Loyola Lima Peru; 3 Red Peruana de Salud Colectiva, Lima, Peru. Red Peruana de Salud Colectiva Lima Peru

**Keywords:** COVID-19, SARS-CoV-2, Severity of Illness Index, Pregnancy, Newborn.

## Abstract

**Objectives.:**

To determine the association between symptoms of COVID-19 infection and adverse maternal-perinatal outcomes in pregnant women from a referral hospital.

**Materials and methods.:**

Analytical cross-sectional study of women in the third trimester of pregnancy hospitalized due to COVID-19 in the gynecology and obstetrics area of a general hospital in Lima during 2020. Clinical and obstetric variables were collected. Fisher’s exact test and Chi-square test were used during the descriptive analysis. Poisson regression was used to find the association between the variables of interest, with a 95% confidence interval (95%CI).

**Results.:**

A total of 272 pregnant women were included, 50.3% of whom had symptoms of infection. Of these, 35.7% of the pregnant women and 16.5% of the newborns had an adverse outcome. Having symptoms of COVID-19 infection increased the risk of maternal complications as a whole (PR= 2.32 95%CI: 1.61-3.34), premature rupture of membranes (PR= 2.73 95%CI: 1.51-4.94) and preeclampsia (PR= 2.73 95%CI: 1.51-4.94). Similarly, symptoms of COVID-19 infection increased the risk of perinatal complications as a whole (PR= 2.51 95%CI: 1.34-4.68) and acute fetal distress (PR= 2.99 95%CI: 1.07-8.38).

**Conclusions.:**

The presence of symptoms of COVID-19 infection increase the risk of adverse maternal-perinatal outcomes.

## INTRODUCTION

The COVID-19 pandemic has caused more than 615 million confirmed cases and more than 6.5 million deaths worldwide ^1^. In Peru, despite initial containment measures, there were more than four million cases, with Metropolitan Lima, Arequipa and Piura being the departments with the most confirmed cases, and Lima, Piura and La Libertad having the highest mortality rates ^2^.

The clinical manifestations of patients infected with COVID-19 occur approximately 7 to 14 days after the infection ^3^. Flu-like symptoms are the most common ones and may progress to severe illness with respiratory failure and the need for mechanical ventilation, admission to the intensive care unit or death ^3^. The Peruvian Ministry of Health (MINSA) classifies COVID-19 infection by severity: asymptomatic, mild, moderate and severe ^4^. This classification can be applied to vulnerable patients, such as pregnant women.

Due to metabolic and physiological changes, pregnant women may present alterations in the immune system, making them more susceptible to COVID-19 ^5^. Immunological adaptations have been described, such as a decrease in the number of CD4+, CD8+ and natural killer lymphocytes, which could be associated with a lower response to viral infections such as SARS-CoV-2 ^6^. It has also been reported that pregnant women with symptoms of COVID-19 infection are more prone to maternal and perinatal complications ^7^.

Several systematic reviews have described that pregnant women with COVID-19 have higher maternal mortality rates ^8^, greater maternal stress ^8^, ruptured ectopic pregnancies ^9^, postpartum hemorrhage ^10^, cesarean deliveries ^8^^,^^10^^-^^12^, preterm delivery ^8^^,^^10^^-^^12^ and premature rupture of membranes ^12^. Likewise, abnormal APGAR scores ^9^, neonatal asphyxia ^9^, stillbirth ^8^^,^^9^, fetal death ^8^ and low birth weight ^10^ were found in children born to infected mothers. In Peru, several studies have shown similar results ^13^^-^^15^. These findings raise the hypothesis that symptoms in COVID-19-infected pregnant women may be associated with adverse maternal and perinatal outcomes.

Although several studies have assessed the association between severe symptoms of infection and adverse maternal-perinatal outcomes ^16^^,^^17^, none have evaluated whether differences exist depending on the presence or absence of infection symptoms. Considering that most infected pregnant women are asymptomatic ^10^, the results of a study that evaluates this hypothesis could reinforce the evidence on the need to prioritize the care of pregnant women in a health system with structural problems, such as the Peruvian one ^18^. Therefore, this study aimed to determine the association between symptoms of COVID-19 infection and adverse maternal-perinatal outcomes in a referral hospital.

**KEY MESSAGES**

Motivation for the study. It is necessary to explore the complications of COVID-19-infected pregnant women and their newborns, whose vulnerability as a risk group increased during the SARS-CoV-2 pandemic.

Main findings. Mothers with COVID-19 symptoms and their newborns had more adverse outcomes.

Implications.Our findings are useful to consider improvements in clinical care and timely referral of infected pregnant women.

## MATERIALS AND METHODS

### Design and population

We carried out an observational, analytical and cross-sectional study. The population consisted of pregnant women diagnosed with COVID-19 who were admitted to the emergency department of the Gynecology Unit of the Santa Rosa Hospital from January 2020 to January 2021. The unit of analysis was the medical records of the pregnant women and their newborns. This is a highly complex hospital that provides outpatient and specialized care ^19^.

All pregnant women in labor over 18 years of age diagnosed with SARS-CoV-2 infection by IgG/IgM serological tests and their newborns were included. We used these tests because they were the standard of medical care in that hospital during the study period. The medical records of pregnant women with incomplete or illegible information were excluded.

### Sample

We included all pregnant women (n=272) diagnosed with symptoms of COVID-19 infection and all 272 neonates born of these pregnancies during the study period. In order to calculate the statistical power, the prevalence of prematurity (10%) in the general population reported in the 2019 Peruvian epidemiological bulletin was used as a proxy for neonatal complications, as well as an approximation of pregnant women with non-severe symptoms of COVID-19 infection ^36^. Likewise, we established a severity probability of 255 for pregnant women with symptoms of COVID-19 infection, so that the sample size ratio between the symptomatic and asymptomatic groups was three. We included 272 pregnant women in the study. The statistical power was calculated with these parameters and a confidence level of 95% in the EPIDAT version 4.2 statistical software. As a result, we obtained a statistical power of 99.5% to report prevalence ratios of 3.3 or more.

### Variables

The exposure variable was the presence of “symptoms of COVID-19 infection” and was dichotomized into asymptomatic and symptomatic. This variable, like all the others, was not assessed primarily based on symptoms, but was obtained from the medical records. The attending physician registered the data in the medical records.

Two unweighted indices were included as dichotomized categorical outcome variables on an exploratory basis: adverse maternal outcomes that included at least one adverse maternal outcome and adverse perinatal outcomes that included at least one adverse perinatal outcome. Maternal adverse outcomes included premature rupture of membrane, postpartum hemorrhage, and preeclampsia, as found in the medical records. Perinatal adverse outcomes included preterm delivery, low birth weight (less than 2500g), acute fetal distress, perinatal death (if death occurred between 22 weeks of gestation and 28 days after birth) and Apgar score <7 at one minute and five minutes ^9^.

Demographic variables were included, such as maternal age in ranges and gestational age, measured in weeks and grouped according to the prematurity classification with a cut-off point of 37 weeks of gestation. We also included clinical variables such as the number of pregnancies (first and multiple), and complete prenatal controls (greater than or equal to six prenatal controls). All variables were measured by the attending physician (specialist in gynecology and obstetrics) and were registered in the medical records.

### Procedure

After obtaining ethical approval, we visited the Santa Rosa Hospital between 12/06/2021 and 12/13/2021. We were granted physical access to the medical records of patients who were admitted to hospital to give birth in the obstetrics unit during 2020. The information was collected on a sheet of paper which was then used to complete our database. The exposure variable was obtained from the COVID-19 screening test on the date of admission to the obstetrics emergency department. Maternal clinical and laboratory variables were measured at admission to the emergency department and during the postnatal period. Newborn clinical and laboratory variables were measured in the neonatology service.

### Statistical analysis

Statistical analysis was carried out in the STATA/SE v16 ® software (Texas, USA). Absolute and relative frequencies were used for descriptive analysis because all variables were categorical. Bivariate analysis was carried out with Fisher’s exact test and Chi-square test, depending on the assumptions. For the multivariate analysis, we used the Poisson family generalized linear model with log link function and robust variance to calculate crude and adjusted prevalence ratios (PR) with 95% confidence intervals (95%CI). In the adjusted model we included variables that had a value of p<0.05 in the crude model. The epidemiological model included theoretical confounding variables such as maternal age ^20^^,^^21^, gestations ^22^, prenatal controls ^23^ and gestational age ^24^, as described in the reviewed literature.

### Ethical Aspects

The research protocol was approved by the Ethics and Research Subcommittee of the Faculty of Health Sciences of the Peruvian University of Applied Sciences (UPC) by resolution FCS-SCE/1335-12-21 and was exempted from ethical review because it used information from medical records. It was also approved by the Methodological Committee of the Santa Rosa Hospital by resolution N° 138-2020-DG-HSR-MINSA. It was also registered in the Health Research Projects Platform (PRISA) of the Instituto Nacional de Salud (INS) under code EI00002571.

## RESULTS

During 2020, 272 pregnant women in labor were admitted to Santa Rosa Hospital and diagnosed with COVID-19, of whom 50.3% had symptoms of infection. Of all the pregnant women, 89.3% did not have complete prenatal controls, 35.7% had an adverse maternal outcome and the most frequent maternal complication was premature rupture of membranes. Of the 272 newborns born to infected pregnant women, 16.5% had adverse outcomes, the most frequent being acute fetal distress (7%). Other general characteristics are shown in Table 1.

**Table 1:** Association between the presence of symptoms in COVID-19-infected pregnant women and their general characteristics.

Characteristics	Total		Symptomatic		Asymptomatic		p-value
	n	%	n	%	n	%	
Demographic variables							
Mother’s age							0.380 ^a^
18-25	107	39.3	48	35.6	59	43.1	
26-35	117	43.0	60	44.4	57	41.6	
36-45	48	17.7	27	20.0	21	15.3	
Gestational age							0.030 ^a^
Preterm	11	4.0	9	6.7	2	1.5	
Full term	261	96.0	126	93.3	135	98.5	
Clinical variables							
Gestations							0.970 ^a^
Multiple	171	62.9	85	63.0	86	62.8	
First	101	37.1	50	37.0	51	37.2	
Prenatal controls							0.530 ^a^
Incomplete	29	10.7	119	88.1	124	90.5	
Complete	243	89.3	16	11.9	13	9.5	
Maternal complications							<0.001 ^a^
Yes	97	35.7	68	50.3	29	21.2	
No	175	64.3	67	49.7	108	78.8	
Premature rupture of membranes							<0.001 ^a^
Yes	48	17.7	35	25.9	13	9.5	
No	224	82.3	100	74.1	124	90.5	
Preeclampsia							0.006 ^a^
Yes	42	15.4	29	21.5	13	9.5	
No	230	84.6	106	78.5	124	90.5	
Postpartum hemorrhage							0.136 ^a^
Yes	11	4.0	8	5.9	3	2.2	
No	261	96.0	127	94.1	134	97.8	
Perinatal complications							<0.001 ^a^
Yes	45	16.5	34	25.2	11	8.0	
No	227	83.5	101	74.8	126	92.0	
Apgar < 7 at Min 1							0.050 ^b^
Yes	13	4.8	10	7.4	3	2.2	
No	259	95.2	125	92.6	134	97.8	
Apgar < 7 at Min 5							0.621 ^b^
Yes	3	1.1	2	1.5	1	0.7	
No	269	98.9	133	98.5	136	99.3	
Prematurity							0.034 ^b^
Yes	11	4.0	9	6.7	2	1.5	
No	261	96.0	126	93.3	135	98.5	
Low birth weight							0.011 ^b^
Yes	13	4.8	11	8.1	2	1.5	
No	259	95.2	124	91.9	135	98.5	
Acute fetal distress							0.034 ^b^
Yes	19	7.0	14	10.4	5	3.7	
No	253	93.0	121	89.6	132	96.3	
Perinatal death							0.245 ^b^
Yes	2	0.7	2	1.5	0	0.0	
No	270	99.3	133	98.5	137	100.0	

a Chi-square test, ^b^ Fisher’s exact test.

During the bivariate analysis between symptoms of COVID-19 infection and adverse maternal outcomes, we found significant differences between premature rupture of membranes (p<0.001) and postpartum hemorrhage (p=0.010) (Table 1). Significant differences were found between adverse perinatal outcomes and having an Apgar <7 at 1 minute (p=0.040), low birth weight (p=0.010), prematurity (p=0.029) and acute fetal distress (p=0.030) (Table 1). While individually, not all adverse outcomes were found to be associated with symptoms of COVID-19 infection; when grouped together, we found an association between being symptomatic with adverse maternal (p<0.001) and adverse perinatal outcomes (p<0.001) (Table 1).

The adjusted multivariate analysis showed that symptoms of COVID-19 infection increased the risk of maternal complications as a whole (PR= 2.32 95%CI:1.61-3.34). The disaggregated analysis showed that COVID-19 infection increased the risk of premature rupture of membranes (PR= 2.73 95%CI:1.51-4.94), and preeclampsia (PR= 2.73 95%CI: 1.51-4.94) (Table 2). Similarly, symptoms of COVID-19 infection were found to increase the risk of perinatal complications as a whole (PR= 2.51 95%CI: 1.34-4.68), and in the disaggregated analysis, it increased the risk of acute fetal distress (PR=2.99 95%CI: 1.07-8.38) (Table 3).

**Table 2:** Association between the presence of symptoms of COVID-19 infection in pregnant women and adverse maternal outcomes.

COVID-19 infection	Maternal complications	p-value	Premature rupture of membranes	p-value	Preeclampsia	p-value	Postpartum hemorrhage	p-value
	PR (95%CI)		PR (95%CI)		PR (95%CI)		PR (95%CI)	
Crude model								
Asymptomatic	Reference		Reference		Reference		Reference	
Symptomatic	2.38 (1.65-3.42)	<0.001	2.73 (1.51-4.94)	0.001	2.26 (1.23-4.16)	0.009	2.71 (0.73-10.01)	0.136
Adjusted model ^a^								
Asymptomatic	Reference		Reference		Reference		Reference	
Symptomatic	2.32 (1.61-3.34)	<0.001	2.73 (1.51-4.94)	0.001	2.73 (1.51-4.94)	0.015	3.07 (0.86-11.07)	0.085

PR: prevalence ratio; 95%CI: 95% confidence interval.

a Model adjusted for maternal age, gestations, prenatal controls and gestational age.

**Table 3:** Association between the presence of symptoms of COVID-19 infection in pregnant women and adverse perinatal outcomes.

COVID-19 infection	Perinatal complications	p-value	Prematurity	p-value	Low weight	p-value	Acute fetal distress	p-value
	PR (95%CI)		PR (95%CI)		PR (95%CI)		PR (95%CI)	
Crude model								
Asymptomatic	Reference		Reference		Reference		Reference	
Symptomatic	3.14 (1.66-5.94)	<0.001	4.57 (1.01-20.81)	0.050	5.58 (1.26-24.78)	0.024	2.84 (1.05-7.68)	0.040
Adjusted model ^a^								
Asymptomatic	Reference		Reference		Reference		Reference	
Symptomatic	2.51 (1.34-4.68)	0.004	1.00 (0.99-1.00)	1.000	3.54 (0.77-16.25)	0.104	2.99 (1.07-8.38)	0.036

PR: prevalence ratio; 95%CI: 95% confidence interval.

a Model adjusted for maternal age, gestations, prenatal controls and gestational age.

## DISCUSSION

Our results show that 3 out of 10 pregnant women with symptoms of COVID-19 infection had adverse maternal outcomes and 2 out of 10 newborns of infected mothers had adverse perinatal outcomes. Having symptoms of COVID-19 infection nearly tripled the risk of preterm rupture of membranes and preeclampsia. Likewise, it tripled the risk of acute fetal distress.

We found a lower proportion of adverse maternal-perinatal outcomes compared to a Peruvian study that reported 48.8% of obstetric complications ^13^ and a study in Kuwait that reported that 26.6% of pregnant women had preterm delivery ^25^. In contrast, our results are similar to those of a systematic review that reported that, among pregnant women with symptoms of COVID-19 infection, 18% had preterm delivery, 19% had low birth weight and 14% had fetal distress ^32^. The differences may be associated with the way in which the variables were defined and the way in which the study samples were selected, resulting in varying prevalence. For example, the Peruvian study ^26^ classified the Apgar variable according to different scores to determine depression in the newborn (≤5 points), whereas we used scores <7 as a cut-off point. It is important to point out that, at the beginning of the pandemic, Peru had a collapsed health system, where structural deficiencies did not improve significantly during the pandemic ^18^; this, together with mobility restrictions, probably caused poor prenatal control and, therefore, increased the risk of maternal-perinatal complications. Nevertheless, it is possible that following national recommendations on the management of pregnant women during the pandemic could have had a positive effect ^33^.

The association between symptoms of COVID-19 infection and maternal-perinatal complications was significant. These results are similar to a systematic review of 42 studies that found that symptomatic pregnant women had an increased risk of preterm delivery when compared to asymptomatic pregnant woman ^16^. Likewise, a multinational cohort study conducted in 18 countries found that gestational age at delivery was 0.8 weeks lower in symptomatic than in asymptomatic pregnant women ^27^. Overall, the presence of any symptom increased the association with adverse outcomes.

Although asymptomatic women diagnosed with COVID-19 infection were at limited risk for most outcomes, there was still an association with preeclampsia ^27^. The presence of fever and shortness of breath, alone or in combination with any group of symptoms, was strongly associated with the risk of preterm delivery ^27^. Another study in the United States found that, compared with asymptomatic patients, adverse perinatal outcomes were more frequent among pregnant women with severe disease, including hypertensive disorders of pregnancy and preterm delivery ^28^. In contrast, mild to moderate infection was not associated with adverse perinatal outcomes compared with asymptomatic patients ^28^.

Although the reasons for the association between symptoms of COVID-19 infection and adverse maternal-perinatal outcomes are unclear, it may be related to the role of immune cell imbalance in symptomatic cases, particularly in severe cases ^29^. During normal pregnancy, the ratio of regulatory T lymphocytes (Treg) to T-helper 17 (Th17) lymphocytes shifts towards Treg cells, which proliferate systematically. Several studies mention that the decrease in the number of Treg cells as well as the increase in Th17 cells are associated with pregnancy complications such as miscarriage, preeclampsia and preterm labor ^30^. Dysregulation of these two cells towards an increase in Th17 cells has been described in patients with symptoms of COVID-19 infection, resulting in uncontrolled systemic inflammation, which worsens with the severity of the infection ^29^. SARS-CoV-2 infection may cause a systemic inflammatory response associated with the pathogenesis of preterm delivery or a suboptimal environment for fetal growth and development ^16^. Similarly, hypercoagulability is associated with the occurrence of preeclampsia ^34^. Additionally, poor placental vascular perfusion has also been reported by placental histopathological studies in patients with symptoms of COVID-19 infection at the time of delivery, which may contribute to poor growth, fetal death or premature delivery or hypertensive disorders ^16^^,^^31^. In that regard, a quasi-experimental study in the Netherlands found that symptom mitigation measures for COVID-19 infection were associated with a decrease in the incidence of preterm delivery ^35^. However, by mechanisms not entirely understood, symptoms are not associated with all complications; thus, a systematic review did not find symptoms of COVID-19 infection associated with gestational diabetes ^16^.

Although cases and mortality due to COVID-19 have decreased, this infection will persist in the community. In this sense, our results could influence medical care and referral strategies in the case of infected pregnant women.

This research has some limitations. First, the cross-sectional study was developed in 2020 during the first wave of the pandemic; therefore, the effect that new variants may have on the presence of symptoms in infected pregnant women was not considered. On the other hand, the lack of control of socioeconomic variables could affect some maternal outcomes such as postpartum hemorrhage, prematurity or low birth weight. Besides, the diagnosis of most patients was made with IgG/IgM immunological tests which, although not the current standard, was recommended by MINSA during 2020, which may alter the prevalence of infected pregnant women and the frequency of their complications. The data were obtained from medical records filled out by hand by the attending physician, so there may be omissions at the discretion of the evaluator. Finally, the study was carried out in a MINSA hospital, so its results cannot be generalized to other institutions in the country. However, one of the strengths of this study is that it was carried out in a category III national referral hospital with comprehensive obstetric care.

In conclusion, symptoms of COVID-19 infection in infected pregnant women and their newborns were associated with a higher probability of developing adverse outcomes in a referral hospital in Peru. Strategies regarding prevention and timely detection of COVID-19 symptoms in pregnant women should be improved. In addition, it is necessary for health personnel to continue monitoring the newborns of infected pregnant women before, during and after delivery, due to the high risk of complications.
