# Supplementary material for: Association between symptoms of COVID-19 infection and adverse maternal-perinatal outcomes in pregnant women at a referral hospital
Source: Rev Peru Med Exp Salud Publica. 2023 Mar 28;40(1):34–41. doi: 10.17843/rpmesp.2023.401.11205 (PMC10953637; doi:10.17843/rpmesp.2023.401.11205)
Supplement: Translation

# Translation: Asociación entre los síntomas de la infección por la COVID-19 y los resultados materno-perinatales adversos en gestantes de un hospital de referencia

**Authors:** Kevyn Angulo-Fernandez, Adrian Olivera-Rojas, Benoît Mougenot, Percy Herrera-Añazco

**Affiliations:** 1 Universidad Peruana de Ciencias Aplicadas, Lima, Perú.; 2 Centro de Excelencia en Investigaciones Económicas y Sociales en Salud, Facultad de Ciencias Empresariales, Universidad San Ignacio de Loyola, Lima, Perú.; 3 Red Peruana de Salud Colectiva, Lima, Perú.

**Keywords:** COVID-19, SARS-CoV-2, Índice de Severidad de la Enfermedad, Embarazo, Recién Nacido

## Abstract

**Objetivos.:**

Determinar la asociación entre los síntomas de la infección por la COVID-19 y los resultados materno-perinatales adversos en gestantes de un hospital de referencia.

**Materiales y métodos.:**

Estudio transversal analítico en gestantes en el tercer trimestre, hospitalizadas por la COVID-19 en el área de ginecología y obstetricia de un hospital general de Lima, en el 2020. Se recogieron variables clínicas y obstétricas. Para el análisis descriptivo se empleó la prueba exacta de Fisher y Chi Cuadrado, y para hallar la asociación entre las variables de interés se usó la regresión de Poisson, con un intervalo de confianza al 95% (IC95%).

**Resultados.:**

Se incluyeron a 272 gestantes, de ellas el 50,3% tuvieron síntomas de infección. El 35,7% de las gestantes y el 16,5% de los recién nacidos tuvieron un resultado adverso. Tener síntomas de la infección por la COVID-19 aumentó el riesgo de complicaciones maternas en su conjunto (RP= 2,32 IC95%: 1,61-3,34), de ruptura prematura de membranas (RP= 2,73 IC95%: 1,51-4,94) y de preeclampsia (RP= 2,73 IC95%: 1,51-4,94). De igual forma, aumentó el riesgo de complicaciones perinatales en su conjunto (RP= 2,51 IC95%: 1,34-4,68) y de sufrimiento fetal agudo (RP= 2,99 IC95%: 1,07-8,38).

**Conclusiones.:**

Los síntomas de la infección por la COVID-19 aumentan el riesgo de resultados materno-perinatales adversos.

## INTRODUCCIÓN

La pandemia por la COVID-19 ha ocasionado más de 615 millones de casos confirmados y más de 6,5 millones de muertes en todo el mundo ^1^. En Perú, a pesar de las medidas iniciales de contención, hubo más de cuatro millones de casos, siendo Lima Metropolitana, Arequipa y Piura, los departamentos con más casos confirmados, y Lima, Piura y La Libertad, los que tuvieron mayor mortalidad ^2^.

Las manifestaciones clínicas de los pacientes infectados por la COVID-19, ocurren aproximadamente a los 7 y 14 días después del contagio ^3^. El síntoma más común se caracteriza por un síndrome gripal que puede progresar a un cuadro grave con insuficiencia respiratoria y la necesidad de ventilación mecánica, ingreso a unidad de cuidados intensivos o fallecimiento ^3^. El Ministerio de Salud (MINSA) del Perú clasifica la infección de la COVID-19 en niveles de severidad: asintomática, leve, moderada y severa ^4^. Esta clasificación puede ser aplicada a pacientes vulnerables, como las gestantes.

En las gestantes, debido a los cambios metabólicos y fisiológicos, pueden ocurrir alteraciones en el sistema inmune y ser más propensas a la COVID-19 ^(^^5^. Se han descrito adaptaciones inmunológicas tales como disminución en el número de linfocitos CD4+, CD8+ y asesinos naturales, que podría asociarse con una menor respuesta frente a infecciones virales como el SARS-CoV-2 ^6^. Asimismo, se ha descrito que las gestantes con síntomas de infección por la COVID-19 son más propensas a complicaciones materno-perinatales ^7^.

Diversas revisiones sistemáticas han descrito que las gestantes con la COVID-19 presentan mayor mortalidad materna ^8^, mayor estrés materno ^8^, embarazos ectópicos rotos ^9^, hemorragia postparto ^10^, partos por cesárea ^8^^,^^10^^-^^12^, parto prematuro ^8^^,^^10^^-^^12^ y rotura prematura de membranas ^12^. Asimismo, en hijos de madres infectadas se encontró puntuaciones de APGAR anormales ^9^, asfixia neonatal ^9^, mortinatalidad ^8^^,^^9^, óbito fetal ^8^ y bajo peso al nacer ^10^. En el Perú, diversos estudios han mostrado resultados similares ^(^^13^^-^^15^. Estos hallazgos plantean la hipótesis de que la presencia de síntomas en las gestantes infectadas por la COVID-19 posiblemente se asocie con resultados materno-perinatales adversos.

Aunque diversos estudios investigaron una asociación entre síntomas severos de la infección y resultados materno-perinatales adversos ^16^^,^^17^, ninguno evaluó si existen diferencias dependiendo de la presencia o no de síntomas de la infección. Considerando que la mayoría de las gestantes infectadas son asintomáticas ^10^, los resultados de un estudio que evalúe esta hipótesis podrían reforzar la evidencia sobre la necesidad de priorizar la atención en las gestantes en un sistema de salud con problemas estructurales, como el peruano ^18^. Por lo expuesto, el objetivo del presente estudio fue determinar la asociación entre los síntomas de infección por la COVID-19 y los resultados materno-perinatales adversos en un hospital de referencia.

**MENSAJES CLAVE**

Motivación para realizar el estudio. Es necesario indagar sobre las complicaciones de las gestantes infectadas por la COVID-19 y sus recién nacidos, cuya vulnerabilidad como grupo de riesgo ha sido señalada durante la pandemia por el SARS-CoV-2.

Principales hallazgos. Se evidenció que las madres sintomáticas por la infección de la COVID-19 y sus recién nacidos tuvieron mayores resultados adversos.

Implicancias. Estos hallazgos son útiles para plantear mejoras en la atención clínica y derivación oportuna de las gestantes infectadas.

## MATERIALES Y MÉTODOS

### Diseño y población

Se realizó un estudio de tipo observacional, analítico y de corte transversal. La población estuvo conformada por las gestantes con diagnóstico de infección por la COVID-19 que ingresaron por el servicio de emergencia en la Unidad de Ginecología del Hospital Santa Rosa, desde enero del 2020 a enero del 2021. La unidad de análisis fueron las historias clínicas de las gestantes y las de sus recién nacidos. Este hospital brinda atención ambulatoria y especializada, siendo un centro de alta complejidad ^19^.

Se incluyeron a todas las gestantes en labor de parto mayores de 18 años con diagnóstico de infección por SARS-CoV-2 mediante pruebas serológicas IgG/IgM y a sus recién nacidos. Estas pruebas fueron empleadas, pues correspondían al estándar de la atención médica en dicho hospital durante el periodo de estudio. Se excluyeron las historias clínicas de las gestantes con información incompleta o ilegible.

### Muestra

Se incluyó al total de gestantes (n=272) con diagnóstico de síntomas de infección por la COVID-19 y a 272 neonatos productos de estas gestaciones en el periodo de estudio. Para el cálculo de la potencia estadística, se utilizó como representante de las complicaciones neonatales a la prevalencia de prematuridad reportada en el boletín epidemiológico peruano del 2019, que reportó una prevalencia de 10% de prematuridad en la población general como una aproximación de las gestantes con síntomas de infección por la COVID-19 no severas ^36^. Así mismo, se estableció una probabilidad de severidad en las gestantes con síntomas de infección por la COVID-19 de 255, con lo que la razón entre tamaños muestrales entre el grupo sintomático y asintomático fue de tres. Se contó con 272 gestantes para desarrollar el estudio. Con estos parámetros y un nivel de confianza del 95%, se procedió a calcular la potencia estadística en el paquete estadístico EPIDAT versión 4.2. Como resultado, se contó con un poder estadístico de 99,5% para reportar razones de prevalencia de 3,3 a más.

### Variables

La variable de exposición fue la presencia de «síntomas de infección por la COVID-19», que se dicotomizó en asintomática y sintomática. La presente variable, al igual que todas las demás, no fue evaluada primariamente a partir de los síntomas, sino que se obtuvo de una ficha presente en la historia clínica. Los datos de esta ficha fueron incluidos por el médico tratante.

En forma exploratoria, se incluyeron dos índices no ponderados como variables de desenlace categóricas dicotomizadas: los resultados maternos adversos que incluyó al menos un desenlace adverso materno y los resultados perinatales adversos que incluyó al menos un desenlace adverso perinatal. Entre los desenlaces adversos maternos se consideraron a la ruptura prematura de membrana, hemorragia posparto y preeclampsia, según estuvieron registradas en las historias clínicas. Entre los desenlaces adversos perinatales se consideraron a la ocurrencia de parto prematuro, bajo peso al nacer (menor de 2500 g), sufrimiento fetal agudo, muerte perinatal (si la muerte ocurrió entre la semana 22 de gestación y 28 días después del nacimiento) y el valor de la prueba de Apgar <7 al minuto y a los cinco minutos ^9^. 

Se incluyeron variables demográficas como la edad de la madre agrupada en rangos de edad y la edad gestacional, determinada en semanas de gestación y agrupada de acuerdo con la clasificación de prematuridad con un punto de corte 37 semanas de gestación, y variables clínicas como el número de gestaciones (primigesta y multigesta), los controles prenatales completos (mayor o igual a seis controles prenatales). Todas las variables fueron determinadas por el médico tratante (especialista en ginecología y obstetricia) y fueron consignadas en la historia clínica.

### Procedimiento

Luego de la aprobación ética se hicieron visitas al área de archivos del Hospital Santa Rosa entre el 06/12/2021 al 13/12/2021, donde se tuvo acceso físico a las historias clínicas de las pacientes que acudieron para recibir atención de parto en el servicio de obstetricia durante el año 2020. La información se recolectó en una ficha en hoja A4 con la se pudo concluir con la elaboración de la base de datos. La variable de exposición se obtuvo del examen de tamizaje de COVID-19 correspondiente a la fecha de ingreso al servicio de emergencia de obstetricia. Para extraer las demás variables se consideraron los parámetros clínicos y de laboratorios correspondientes a la fecha de ingreso de las gestantes por emergencia y durante su evolución posparto, así como los parámetros clínicos y de laboratorios del recién nacido en el servicio de neonatología.

### Análisis estadístico

El análisis estadístico se realizó en el programa STATA/SE v16 **®** (Texas, USA). Debido a que todas las variables fueron categóricas, se usaron frecuencias absolutas y relativas para el análisis descriptivo. Para el análisis bivariado se usó la prueba exacta de Fisher y de Chi cuadrado, según el cumplimiento de los supuestos. Para el análisis multivariado se utilizó el modelo lineal generalizado de familia Poisson con función de enlace logarítmico y varianza robusta, para calcular las razones de prevalencia (RP) crudos y ajustados con intervalos de confianza del 95% (IC95%). Para ingresar al modelo ajustado estadístico se consideraron las variables que tuvieron un valor de p<0,05 en el modelo crudo y para el modelo epidemiológico se incluyeron variables de confusión teóricas como la edad materna ^20^^,^^21^, gestaciones ^22^, controles prenatales ^23^ y edad gestacional ^24^, descritas en la literatura revisada.

### Aspectos éticos

El protocolo de investigación fue aprobado por el Subcomité de Ética e Investigación de la Facultad de Ciencias de la Salud de la Universidad Peruana de Ciencias Aplicadas (UPC) mediante resolución FCS-SCE/1335-12-21 y se exoneró de revisión ética por usar historias clínicas como fuente de datos. También fue aprobado por el Comité Metodológico del Hospital Santa Rosa mediante resolución N° 138-2020-DG-HSR-MINSA. Asimismo, se registró en la Plataforma de Proyectos de Investigación en Salud (PRISA) del Instituto Nacional de Salud (INS) con el código EI00002571. 

## RESULTADOS

Durante el 2020 ingresaron al Hospital Santa Rosa 272 gestantes en labor de parto que fueron diagnosticadas con la COVID-19, de las cuales el 50,3% tuvieron síntomas de la infección. De todas las gestantes, el 89,3% no presentó controles prenatales completos, el 35,7% tuvo un resultado materno adverso y la complicación materna más frecuente fue la rotura prematura de membranas. De los 272 recién nacidos de las gestantes infectadas, el 16,5% tuvieron desenlaces adversos, siendo el más frecuente el sufrimiento fetal agudo (7%). El resto de las características generales se muestran en la Tabla 1.

**Tabla 1:** Asociación entre la presencia de síntomas en gestantes infectadas por la COVID-19 y sus características generales.

Características	Total		Sintomática		Asintomática		Valor de p
	n	%	n	%	n	%	
Variables demográficas							
Edad materna							0,380 ^a^
18-25	107	39,3	48	35,6	59	43,1	
26-35	117	43,0	60	44,4	57	41,6	
36-45	48	17,7	27	20,0	21	15,3	
Edad gestacional							0,030 ^a^
Pretérmino	11	4,0	9	6,7	2	1,5	
Término	261	96,0	126	93,3	135	98,5	
Variables clínicas							
Gestaciones							0,970 ^a^
Multigesta	171	62,9	85	63,0	86	62,8	
Primigesta	101	37,1	50	37,0	51	37,2	
Controles prenatales							0,530 ^a^
Incompleto	29	10,7	119	88,1	124	90,5	
Completo	243	89,3	16	11,9	13	9,5	
Complicaciones maternas							<0,001 ^a^
Sí	97	35,7	68	50,3	29	21,2	
No	175	64,3	67	49,7	108	78,8	
Rotura prematura de membranas							<0,001 ^a^
Sí	48	17,7	35	25,9	13	9,5	
No	224	82,3	100	74,1	124	90,5	
Preeclampsia							0,006 ^a^
Sí	42	15,4	29	21,5	13	9,5	
No	230	84,6	106	78,5	124	90,5	
Hemorragia posparto							0,136 ^a^
Sí	11	4,0	8	5,9	3	2,2	
No	261	96,0	127	94,1	134	97,8	
Complicaciones perinatales							<0,001 ^a^
Sí	45	16,5	34	25,2	11	8,0	
No	227	83.5	101	74,8	126	92,0	
Apgar < 7 Min 1							0,050 ^b^
Sí	13	4,8	10	7,4	3	2,2	
No	259	95,2	125	92,6	134	97,8	
Apgar < 7 Min 5							0,621 ^b^
Sí	3	1,1	2	1,5	1	0,7	
No	269	98,9	133	98,5	136	99,3	
Prematuridad							0,034 ^b^
Sí	11	4,0	9	6,7	2	1,5	
No	261	96,0	126	93,3	135	98,5	
Bajo peso al nacer							0,011 ^b^
Sí	13	4,8	11	8,1	2	1,5	
No	259	95,2	124	91,9	135	98,5	
Sufrimiento fetal agudo							0,034 ^b^
Sí	19	7,0	14	10,4	5	3,7	
No	253	93,0	121	89,6	132	96,3	
Muerte perinatal							0,245 ^b^
Sí	2	0,7	2	1,5	0	0,0	
No	270	99,3	133	98,5	137	100,0	

a Prueba de Chi Cuadrado, ^b^ prueba exacta de Fisher.

En el análisis bivariado, entre los síntomas de infección por la COVID-19 y los resultados maternos adversos, se encontraron diferencias significativas entre la rotura prematura de membranas (p<0,001) y la hemorragia posparto (p=0,010) (Tabla 1). Con los resultados perinatales adversos, también se encontraron diferencias significativas con tener un Apgar <7 al primer minuto (p=0,040), el bajo peso al nacer (p=0,010), la prematuridad (p=0,029) y el sufrimiento fetal agudo (p=0,030) (Tabla 1). Si bien, de forma individual, no todos los resultados adversos resultaron asociados con los síntomas de infección por la COVID-19; cuando se agruparon, se halló una asociación entre ser sintomática con los resultados maternos adversos (p<0,001) y perinatales adversos (p<0,001) (Tabla 1).

En el modelo ajustado del análisis multivariado, se encontró que los síntomas de infección por la COVID-19 aumentaron el riesgo de complicaciones maternas en su conjunto (RP= 2,32 IC95%:1,61-3,34). En el análisis disgregado, se encontró que aumentó el riesgo de ruptura prematura de membranas (RP= 2,73 IC95%:1,51-4,94), y de preeclampsia (RP= 2,73 IC95%: 1,51-4,94) (Tabla 2). De igual forma, se encontró que los síntomas de infección por la COVID-19 aumentaron el riesgo de complicaciones perinatales en su conjunto (RP= 2,51 IC95%: 1,34-4,68), y en el análisis disgregado, aumentó el riesgo de sufrimiento fetal agudo (RP=2,99 IC95%: 1,07-8,38) (Tabla 3).

**Tabla 2:** Asociación entre la presencia de síntomas de infección por la COVID-19 en gestantes y los resultados maternos adversos.

Infección por la COVID-19	Complicaciones maternas	Valor de p	Ruptura prematura de membranas	Valor de p	Preeclampsia	Valor de p	Hemorragia posparto	Valor de p
	RP (IC95%)		RP (IC95%)		RP (IC95%)		RP (IC95%)	
Modelo crudo								
Asintomática	Referencia		Referencia		Referencia		Referencia	
Sintomática	2,38 (1,65-3,42)	<0,001	2,73 (1,51-4,94)	0,001	2,26 (1,23-4,16)	0,009	2,71 (0,73-10,01)	0,136
Modelo ajustado ^a^								
Asintomática	Referencia		Referencia		Referencia		Referencia	
Sintomática	2,32 (1,61-3,34)	<0,001	2,73 (1,51-4,94)	0,001	2,73 (1,51-4,94)	0,015	3,07 (0,86-11,07)	0,085

RP: razón de prevalencia, IC95%: intervalo de confianza al 95%.

a Modelo ajustado por edad materna, gestaciones, controles prenatales y edad gestacional.

**Tabla 3:** Asociación entre la presencia de síntomas de infección por la COVID-19 en gestantes y los resultados perinatales adversos.

Infección por la COVID-19	Complicaciones perinatales	Valor de p	Prematuridad	Valor de p	Bajo peso	Valor de p	Sufrimiento fetal agudo	Valor de p
	RP (IC95%)		RP (IC95%)		RP (IC95%)		RP (IC95%)	
Modelo crudo								
Asintomática	Referencia		Referencia		Referencia		Referencia	
Sintomática	3,14 (1,66-5,94)	<0,001	4,57 (1,01-20,81)	0,050	5,58 (1,26-24,78)	0,024	2,84 (1,05-7,68)	0,040
Modelo ajustado ^a^								
Asintomática	Referencia		Referencia		Referencia		Referencia	
Sintomática	2,51 (1,34-4,68)	0,004	1,00 (0,99-1,00)	1,000	3,54 (0,77-16,25)	0,104	2,99 (1,07-8,38)	0,036

RP: razón de prevalencia, IC95%: intervalo de confianza al 95%.

a Modelo ajustado por edad materna, gestaciones, controles prenatales y edad gestacional.

## DISCUSIÓN

Los principales resultados de la investigación muestran que 3 de cada 10 gestantes con síntomas de infección por la COVID-19 tuvieron resultados maternos adversos y que 2 de cada 10 recién nacidos de madres infectadas tuvieron resultados perinatales adversos. Tener síntomas de infección por la COVID-19 casi triplicó el riesgo de tener ruptura prematura de membranas y de preeclampsia. De igual forma, triplicó el riesgo de sufrimiento fetal agudo.

Se encontró una proporción de resultados materno-perinatales adversos menor a un estudio peruano que reportó un 48,8% de complicaciones obstétricas ^13^ y de un estudio en Kuwait, donde un 26,6% de las gestantes tuvo un parto pretérmino ^25^. Por el contrario, es similar a los resultados de una revisión sistemática que encontró que, entre mujeres embarazadas diagnosticadas con síntomas de infección por la COVID-19, el 18% tuvo parto prematuro, 19% bajo peso al nacer y 14% presentó sufrimiento fetal ^32^. Las diferencias pueden estar asociadas a la forma en que se definieron las variables y a la forma de seleccionar las muestras de los estudios; dando como resultado que la prevalencia varíe. Por ejemplo, en el estudio peruano ^26^, la variable Apgar se clasificó según diferentes puntajes para determinar depresión en el recién nacido (≤5 puntos), mientras que para el presente estudio, el punto de corte fue <7 puntos. Es necesario puntualizar que, al inicio de la pandemia, el Perú contaba con un sistema de salud colapsado, donde las deficiencias estructurales no mejoraron significativamente durante la pandemia ^18^, lo que, junto a las restricciones de movilidad, ocasionaron probablemente un mal control prenatal y, por lo tanto, aumentó el riesgo de mayores complicaciones materno-perinatales. A pesar de eso, es posible que, el seguimiento de las recomendaciones nacionales sobre el manejo de las gestantes durante la pandemia pudo tener un efecto positivo ^33^. 

Se encontró una asociación significativa entre los síntomas de infección por la COVID-19 y las complicaciones materno-perinatales. Estos resultados son similares a una revisión sistemática de 42 estudios que encontró que, en comparación con una gestante asintomática, una gestante sintomática se asoció con un mayor riesgo de parto prematuro ^16^. Asimismo, un estudio de cohorte multinacional realizado en 18 países encontró que, la edad gestacional al momento del parto, fue de 0,8 semanas menor en las gestantes sintomáticas que en las no sintomáticas ^27^. En general, la presencia de cualquier síntoma aumentó la asociación con resultados adversos. 

Aunque las mujeres asintomáticas con diagnóstico de infección por la COVID-19 tenían un riesgo limitado para la mayoría de los resultados, aún existía una asociación con la preeclampsia ^27^. La presencia de fiebre y dificultad para respirar, por separado o en combinación con cualquier grupo de síntomas, se asoció marcadamente con un riesgo de parto prematuro ^27^. Otro estudio en Estados Unidos encontró que, comparado con las pacientes asintomáticas, los resultados perinatales adversos fueron más frecuentes entre las gestantes con enfermedad grave, incluido trastornos hipertensivos del embarazo y parto prematuro ^28^. Por el contrario, la infección leve a moderada no se asoció con resultados perinatales adversos en comparación con las pacientes asintomáticas ^28^.

Aunque las razones de la asociación entre los síntomas de infección por la COVID-19 y los resultados adversos materno-perinatales no son claras, es posible que esté relacionado con el papel del desequilibrio de células inmunes que ocurre en los casos sintomáticos, sobre todo en los casos severos ^29^. Durante el embarazo normal, la relación de linfocitos T reguladores (Treg) y linfocitos T cooperadores 17 (Th17) cambian a favor de las células Treg las cuales proliferan sistemáticamente. Diversos estudios mencionan que la disminución del número de células Treg y el aumento de células Th17 están asociados con complicaciones en el embarazo, como aborto espontaneo, preeclampsia y trabajo de parto prematuro ^30^. En los pacientes con síntomas de infección por la COVID-19 se ha descrito desregulación de estas dos células hacia un aumento de las células Th17 dando como resultado una inflamación sistémica descontrolada, que empeora con la severidad de la infección ^29^. La infección por SARS-CoV-2 puede causar una respuesta inflamatoria sistémica implicada en la patogenia del parto prematuro o un ambiente subóptimo para el crecimiento y desarrollo fetal ^16^. De igual forma, hipercoagulabilidad asociada a la ocurrencia de preeclampsia ^34^. Adicionalmente, también se ha encontrado una mala perfusión vascular placentaria en estudios histopatológicos placentarios en pacientes con síntomas de infección por la COVID-19 en el momento del parto, lo que puede contribuir al pobre crecimiento, muerte fetal o parto prematuro o trastornos hipertensivos ^16^^,^^31^. En ese sentido, un estudio cuasi-experimental en los Países Bajos encontró que las medidas de mitigación de síntomas de infección por la COVID-19 estaban asociadas con una reducción de la incidencia de parto prematuro ^35^. No obstante, por mecanismos no del todo claros, los síntomas no se asocian a todas las complicaciones, así, una revisión sistemática no encontró que los síntomas de la infección por la COVID-19 se asocian a diabetes gestacional ^16^. 

A pesar de que los casos y la mortalidad por la COVID-19 disminuyeron, esta infección aún permanecerá en la sociedad. En ese sentido, estos resultados podrían influir en las estrategias de atención clínica y derivación en caso de gestantes infectadas.

Esta investigación tiene algunas limitaciones. En primer lugar, el estudio de diseño transversal fue desarrollado en el 2020 durante la primera ola de la pandemia, por lo tanto, no se consideró el efecto que nuevas variantes que puedan tener en la presencia de síntomas en las gestantes infectadas. Por otro lado, la falta de control de variables socioeconómicas podría afectar algunos desenlaces maternos como la hemorragia posparto, la prematuridad o el bajo peso al nacer. En segundo lugar, el diagnóstico de la mayoría de los pacientes se hizo con las pruebas inmunológicas IgG/IgM que, aunque no es la forma del estándar actual, fue lo recomendado por el MINSA durante el 2020, lo que puede alterar la prevalencia de gestantes infectadas y la frecuencia de sus complicaciones. En tercer lugar, los datos fueron obtenidos de historias clínicas llenadas a mano por el médico tratante, con lo que pueden existir omisiones según el criterio del evaluador durante la emergencia médica. En cuarto lugar, es un estudio hecho en un hospital del MINSA, por lo que sus resultados no pueden ser generalizables a otras instituciones del país. Sin embargo, una fortaleza del estudio es que se hizo en un hospital de referencia nacional de categoría III con amplia atención obstétrica.

En conclusión, los síntomas de infección por la COVID-19 se asociaron a mayor probabilidad de desarrollar resultados adversos en gestantes infectadas y sus recién nacidos, en un hospital de referencia del Perú. Se sugiere reforzar la prevención y la detección oportuna de los síntomas de infección por la COVID-19 en gestantes. Adicionalmente, es necesario que el personal de salud continúe con la monitorización de los recién nacidos de aquellas gestantes infectadas antes, durante y después del parto, debido al alto riesgo de complicaciones.
